# Strong binding and fluorescence sensing of bisphosphonates by guanidinium-modified calix[5]arene

**DOI:** 10.3762/bjoc.14.157

**Published:** 2018-07-19

**Authors:** Jie Gao, Zhe Zheng, Lin Shi, Si-Qi Wu, Hongwei Sun, Dong-Sheng Guo

**Affiliations:** 1 College of Chemistry, Key Laboratory of Advanced Energy Materials Chemistry (Ministry of Education), Nankai University, Tianjin 300071, China; 2 Collaborative Innovation Center of Chemical Science and Engineering, Nankai University, Tianjin 300071, China

**Keywords:** bisphosphonate, calixarene, fluorescence sensing, macrocyclic chemistry, indicator displacement assay

## Abstract

Based on the indicator displacement assay (IDA) approach, we herein report the fluorescence “switch-on” sensing and quantitative detection of bisphosphonates (BPs), a class of drugs extensively used in the treatment of patients with various skeletal diseases. Guanidinium-modified calix[5]arene (GC5A) affords strong binding on the micromolar to nanomolar level towards BPs dominantly via multiple salt bridge interactions, which was evaluated by fluorescence competitive titrations. Fluorescent IDA enables the highly sensitive and label-free detection of BPs in buffer solution, and more importantly, in artificial urine. Calibration lines were therefore set up in untreated artificial urine, allowing for quantifying the concentrations of BPs in the biologically relevant low range.

## Introduction

**Scheme 1:**
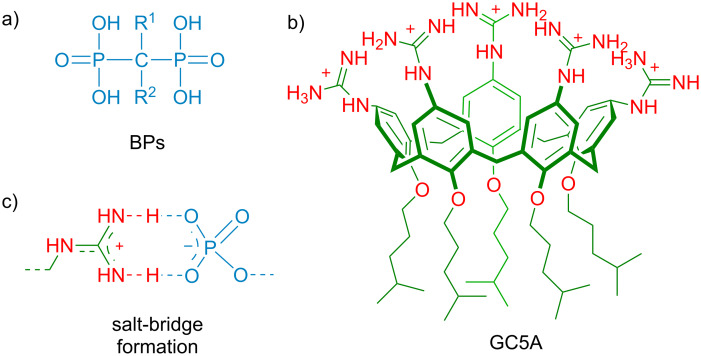
The chemical structures of (a) bisphosphonates (BPs) and (b) guanidinium-modified calix[5]arene (GC5A). (c) Schematic illustration of a salt-bridge between a phosphate anion and a guanidinium cation.

Bisphosphonates (BPs) are a kind of drugs characterized by the −C(PO_3_)_2_ group (Scheme 1a) for treating various types of bone disorders and calcium metabolic diseases [1]. They are widely used in the treatment of osteoporosis, osteitis deformans, hypercalcaemia and bone pain caused by bone metastases of malignant tumors [2–3]. In addition, BPs are increasingly considered due to their potential role in preventing and treating cancer-induced bone loss and antitumor effects [4–5]. With this regard, assays for BPs are significant for identifying the quality of pharmaceutical formulations, as well as monitoring drug plasma concentrations, analyzing drug biodistribution in bone tissue, and detecting drug excretion in urine. For example, BPs are of poor bioavailability if orally administered (generally with absorption less than 1%) and about 50% of the absorbed dose is taken up selectively by the skeleton. Therefore, tracking the concentrations of BPs in biological systems has clinical significance for the doctor to record a patient's drug absorption in real time and adjust the dosage in time [6]. Numerous analytical methods, based on diverse principles, have been developed for detecting BPs in pharmaceuticals and biological materials [7–8]. Due to BPs′ high polarity, they are difficult to separate on reversed-phase columns. To make them more amenable to analysis, ion-pairing or complex-forming reagents were used to decrease the ionic character of the molecules [9]. However, this method will greatly reduce the life of the column [10]. Moreover, the absence of a chromophore in most BPs lead to the employment of derivatization by an UV–vis light-absorbing or fluorescence label for detection [11–12]. However, directly labeling BPs in biological media is difficult because many other components can reduce the efficiency of the labeling reaction. Especially in urine, it is extraordinary challenging to achieve labelling of BPs because urine generally contains a large amount of polar compounds such as phosphates, unless these are removed in advance [7]. Ion chromatography combined with conductivity detection (or other detectors) can be used to solve the problems of separation and detection of BPs, but the relatively expensive instruments often are not affordable [13]. As a result, the development of a label-free optical method for BPs detection is highly appealing in view of low cost, ease of use and high sensitivity of optical sensing modalities, but remains a challenge since BPs are considered as unlabeled analytes.

**Scheme 2:**
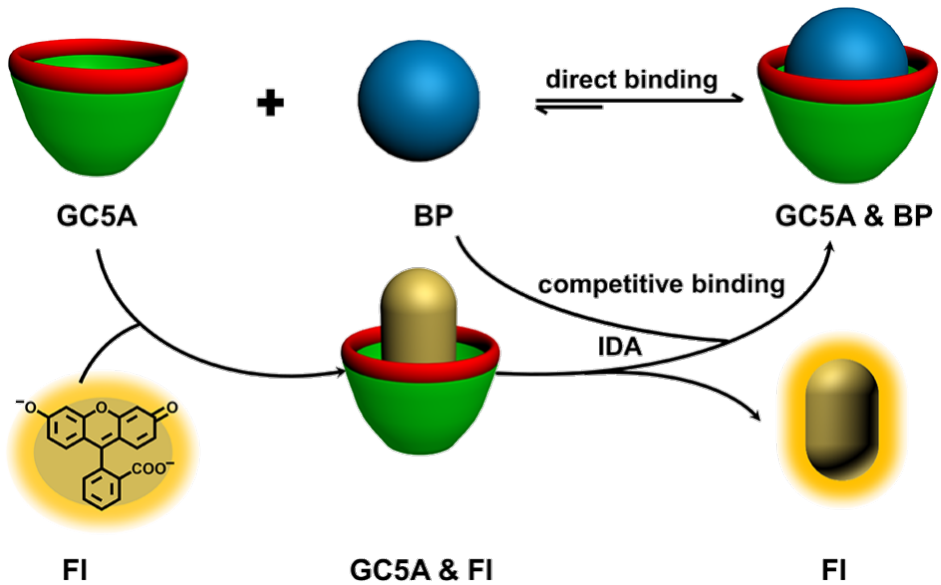
Schematic illustration of the binding between BPs and GC5A and the operating IDA principle of fluorescence “switch-on” sensing of BPs by the GC5A·Fl reporter pair.

With the development of the host–guest concept in supramolecular chemistry, the indicator displacement assay (IDA), pioneered by Prof. Anslyn and co-workers, has been popularized as an alternative strategy for molecular sensing, complementary to direct sensing [14–16]. IDA refers to a signal change of an indicator upon competition between an analyte and the indicator for the binding to a receptor. The label-free method renders IDA particularly suitable for the detection of analytes lacking chromophores. The key factor in IDA is the rational design of artificial receptors that are capable of binding analytes strongly and specifically. Calixarenes are the third generation of macrocyclic receptors after crown ethers and cyclodextrins. Due to their facial modification, Prof. Böhmer demonstrated calixarenes as having “(almost) unlimited possibilities” [17]. We have focused on molecular recognition and self-assembly of water-soluble calixarene derivatives for a long time [18–24], directed by exploring biomedical applications of them [25–28]. In this work, we report a fluorescent IDA approach for detecting BPs quantitatively in not only buffer solution but also artificial urine (Scheme 2). The rationale behind the IDA approach is the strong and selective complexation of BPs by guanidinium-modified calix[5]arene (GC5A, Scheme 1b). Such label-free sensing strategy exhibits potential application in real-time monitoring concentrations of BPs in urine and pharmacokinetic studies.

## Results and Discussion

The main skeleton of BPs possesses two phosphate groups which are potential binding sites and therefore GC5A was tested as binding receptor. GC5A was prepared according to our previous procedure [26] and the guanidinium groups installed in the upper rim are expected to form multiple salt bridge interactions (charge-assisted hydrogen bonds) with the phosphate groups of BPs (Scheme 1c) [26,29].

To execute IDA, we employed fluorescein (Fl) as the reporter dye according to our previously published result [26]. Fl of high brightness is strongly encapsulated into the GC5A cavity (*K*_a_ = 5.0 × 10^6^ M^−1^), accompanied with a drastic complexation-induced fluorescence quenching (*I*_free_/*I*_bound_ = 37). Taken together, these factors make the GC5A·Fl reporter pair an ideal combination for the projected IDA application. IDA was implemented to determine the binding affinities between GC5A and BPs via competitive fluorescence titrations. More importantly, the displacement of the reporter dye, accompanied with fluorescence recovery, offers the opportunity for fluorescence “switch-on” sensing of BPs. In general, fluorescent IDA could be operated at low μM or even nM concentrations, which is desirable with respect to sensing sensitivity.

**Scheme 3:**
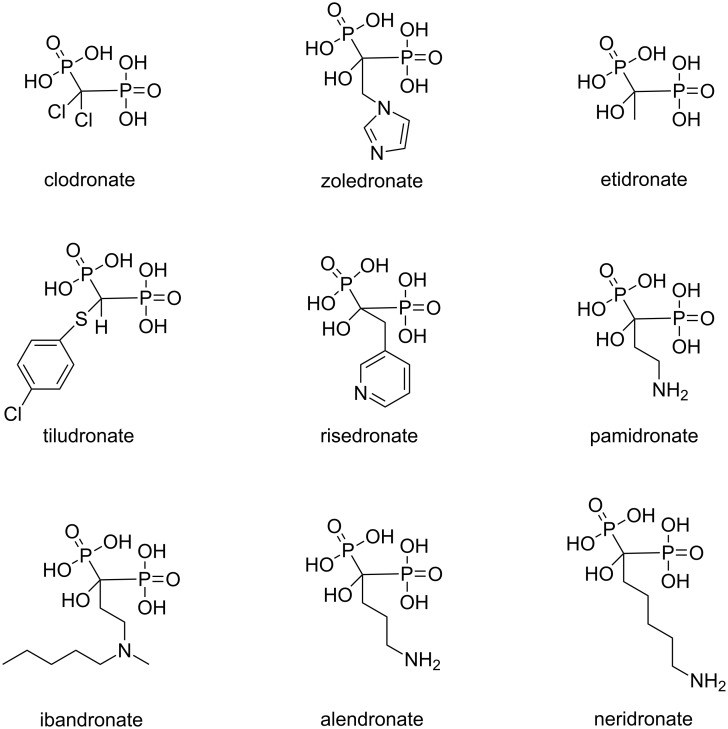
The chemical structures of the selected BP drugs.

**Figure 1:**
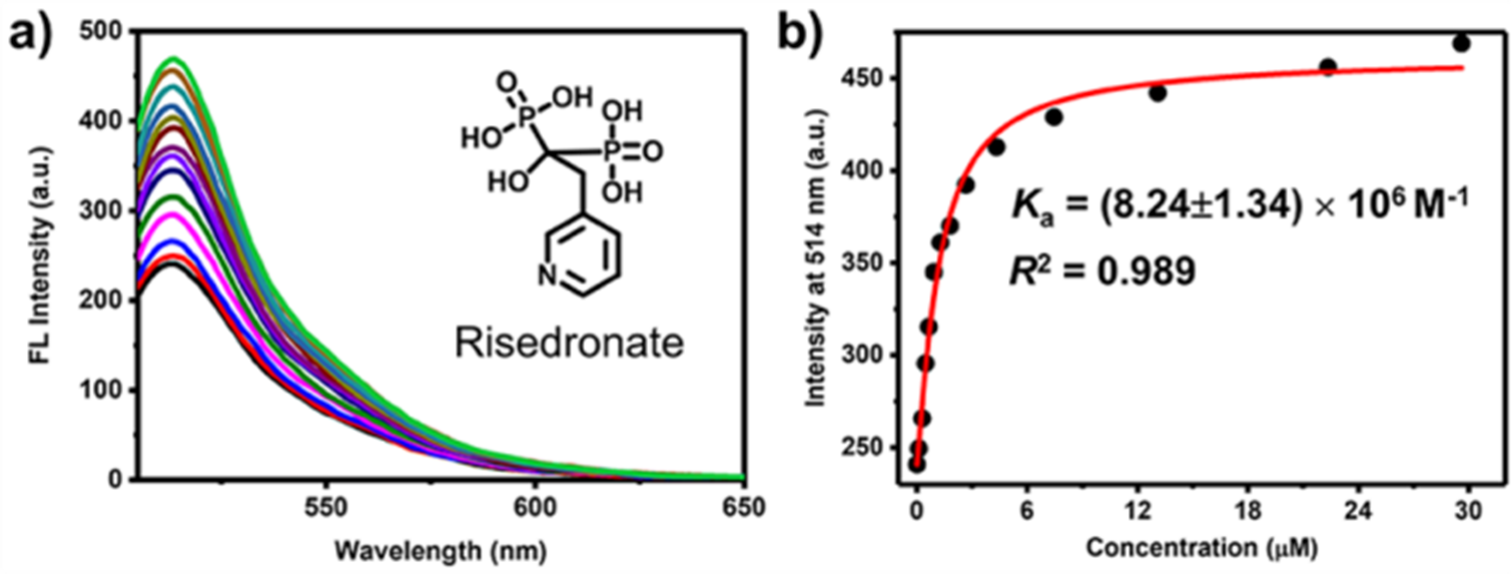
(a) Fluorescence competitive titration of GC5A·Fl (0.9/1.0 μM) with risedronate (up to 29.6 μM) in HEPES buffer (10 mM, pH 7.4) at 25 °C, λ_ex_ = 500 nm. (b) The competitive titration curve, λ_em_ = 514 nm, and fitting data according to a 1:1 competitive binding model.

We tested the host–guest complexation of GC5A with a total number of 9 BP drugs clinically applied (Scheme 3) by utilizing competitive fluorescence titrations. Upon gradual addition of BPs gives rise to the displacement of Fl out of the GC5A cavity, and therefore recovery of the intrinsic emission of Fl (Figure 1 and Figures S1–S8 in Supporting Information File 1). The data fitted well with the 1:1 competitive binding model, giving the *K*_a_ values as listed in Table 1. Ibandronate, alendronate and neridronate gave an around 40–100 times weaker binding than etidronate, which is probably due to the aminoalkyl-substituents present in the former compounds. However, pamidronate having an aminoethyl substituent shows comparable binding to etidronate. At present, the reason for this behavior remains unclear. Overall, the strong binding of 9 BPs with GC5A with association constants in the μM to nM range is suitable for the following sensing study.

**Table 1:** Association constants (*K*_a_) of BPs and GC5A determined according to the competitive titration method. All experiments were performed in HEPES buffer (10 mM, pH 7.4) at 25 °C.

BPs	*K*_a_ (M^−1^)

clodronate	(6.60 ± 0.37) × 10^7^
zoledronate	(1.34 ± 0.12) × 10^7^
etidronate	(1.24 ± 0.15) × 10^7^
tiludronate	(8.31 ± 0.97) × 10^6^
risedronate	(8.24 ± 1.34) × 10^6^
pamidronate	(5.37 ± 0.71) × 10^6^
ibandronate	(3.01 ± 0.65) × 10^5^
alendronate	(1.80 ± 0.18) × 10^5^
neridronate	(1.26 ± 0.26) × 10^5^

**Figure 2:**
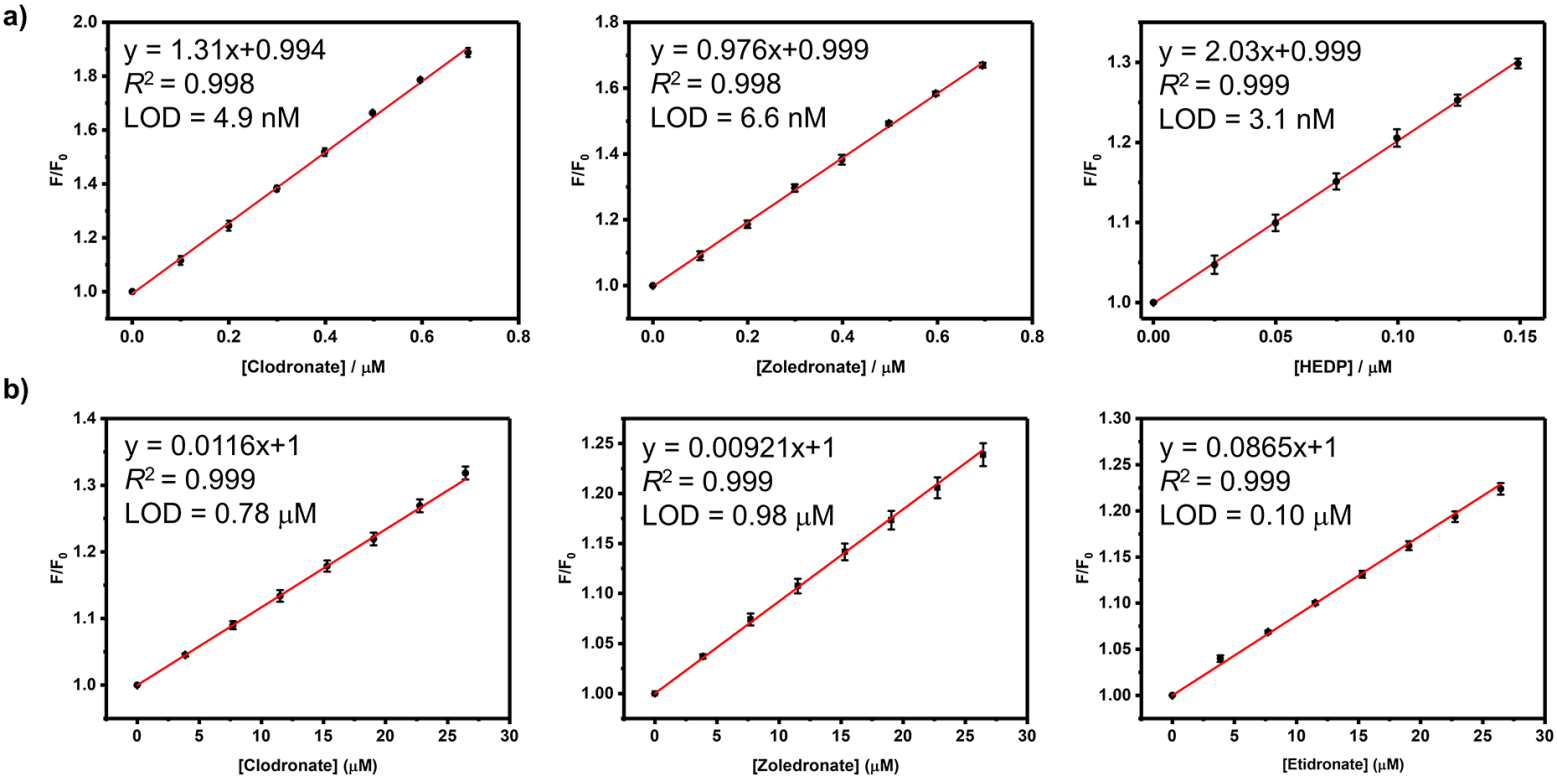
The set-up calibration lines of the fluorescence intensities for quantitatively determining the concentrations of clodronate, zoledronate and etidronate in (a) HEPES buffer (10 mM, pH 7.4) and (b) artificial urine at 25 °C. Error bars could not be shown if less than 0.005.

By executing IDA based on the GC5A·Fl reporter pair, we realized the fluorescence “switch-on” sensing of BPs. We herein selected clodronate, zoledronate and etidronate to further investigate their quantitative detection. As shown in Figure 2a, increasing the concentrations of BPs resulted in a practically linear fluorescence increase. The limit of detection (LOD) for BPs was calculated to be 4.9 nM for clodronate, 6.6 nM for zoledronate and 3.1 nM for etidronate by utilizing a 3σ/slope method [7,30–31]. These low values of LOD also demonstrate the high sensitivity of the IDA strategy based on the GC5A·Fl combination. The ultra-sensitive detection of BPs down to the low nM range benefits from not only the strong binding ability of GC5A but also the efficient fluorescence response of the GC5A·Fl reporter pair.

Compared to sensing in buffered solutions, it is more challenging to detect analytes directly in complex biological samples such as urine, blood serum or plasma, saliva, etc. The aforementioned results in HEPES buffer of high performance encouraged us to further test the IDA strategy in urine. As the urine composition is affected by individual differences, water intake, the time of urination and other factors [32], we herein employed artificial urine as the proof-of-principle sample. Although there are numerous interfering substances in artificial urine [33], we still observed the linear increase in fluorescence of the GC5A·Fl reporter pair upon gradual addition of BPs (Figure 2b). The LOD values in artificial urine were calculated as 0.78 μM for clodronate, 0.98 μM for zoledronate, and 0.10 μM for etidronate. With respect to the requisite detection limit in urine BP concentrations typically observed in patients with bone disease after the administration of BPs [7], the present IDA strategy with such low LOD values represents a sensitive approach for detecting BPs. Based on the linear relationship of good performance (*R*^2^: clodronate 0.999, zoledronate 0.999 and etidronate 0.999), we set up a series of calibration lines of the fluorescence output. These calibration lines of BPs are meaningful for quantitatively tracking drug excretion in urine down to the low μM range.

## Conclusion

In conclusion, we have established an IDA approach based on the GC5A·Fl reporter pair for fluorescence “switch-on” sensing and quantitative detection of BPs. Thanks to the strong binding capabilities of GC5A towards BPs, we realized a highly sensitive and label-free detection of BPs through the fluorescent IDA. For accurately determining unknown concentrations of BPs down to the low μM range of tracking drug excretion, calibration lines were successfully set up in artificial urine. The present study paves a new avenue for detecting BPs in a low-cost, easily to operate, label-free and sensitive way, promising feasible application in tracking drug excretion, studying pharmacokinetic processes, and inspecting pharmaceutical quality.

## Supporting Information

File 1 Experimental part.
