# Supplementary material for: Strong binding and fluorescence sensing of bisphosphonates by guanidinium-modified calix[5]arene
Source: Beilstein J Org Chem. 2018 Jul 19;14:1840–5. doi: 10.3762/bjoc.14.157 (PMC6071700; doi:10.3762/bjoc.14.157)
Supplement: File 1 — Experimental part. [file Beilstein_J_Org_Chem-14-1840-s001.pdf]

Supporting Information
for
Strong binding and fluorescence sensing of bisphosphonates by guanidinium-modified calix[5]arene

Jie Gao[1], Zhe Zheng[1], Lin Shi[1], Si-Qi Wu[1], Hongwei Sun*[1] and Dong-Sheng Guo*[1,2]

Address: [1]College of Chemistry, Key Laboratory of Advanced Energy Materials Chemistry (Ministry of Education), Nankai University, Tianjin 300071, China and [2]Collaborative Innovation Center of Chemical Science and Engineering, Nankai University, Tianjin 300071, China

Email: Hongwei Sun - sunhw@nankai.edu.cn; Dong-Sheng Guo - dshguo@nankai.edu.cn

*Corresponding author

Experimental part

Table of contents

1 General methods and materials S2
1.1 Materials. S2
1.2 Samples. S2
1.3 Instruments. S2
1.4 Statistical analysis. S2
2 Supporting results and experimental raw data S2
2.1 Binding affinities of GC5A with BPs. S2
References S5

1 General methods and materials

1.1 Materials. All reagents and solvents were commercially available and used as received unless otherwise specified. Neridronate and fluorescein disodium salt were purchased from Sigma-Aldrich. Clodronate, risedronate and pamidronate were purchased from Meilunbio. Zoledronate, tiludronate and ibandronate were obtained from Energy Chemical. Alendronate and fluorescein disodium salt were purchased from TCI. Etidronate was purchased from Alfa Aesar. GC5A was synthesized according to the previous literature procedure [1].

1.2 Samples. The HEPES buffer solution pH 7.4 was prepared by dissolving 2.38 g of 4-(2-hydroxyethyl)piperazine-1-ethanesulfonic acid (HEPES) in approximately 900 mL double-distilled water. Adjustment of pH 7.4 was achieved at 25 °C by titration with NaOH and dilution to 1000 mL with double-distilled water. The pH value of the buffer solution was then verified on a pH-meter calibrated with three standard buffer solutions. The artificial urine was prepared according to the previous literature [2].

1.3 Instruments. Steady-state fluorescence measurements were recorded in a conventional quartz cuvette (light path 10 mm) on a Cary Eclipse equipped with a Cary single-cuvette peltier accessory.

1.4 Statistical analysis. Quantitative data were expressed as means (standard deviation).

2 Supporting results and experimental raw data

2.1 Binding affinities of GC5A with BPs.

The complexation of GC5A with BPs is so strong that the direct titrations cannot be fitted very well. Therefore the competitive fluorescence titration method was applied to obtain the binding constants. Fluorescein (Fl) was employed as the reporter dye. The binding stoichiometry and the binding affinity (K_a) between GC5A and Fl had been determined as 1:1 and $(5.0 \pm 1.0) \times 10^6$ M^{-1}, respectively [1].

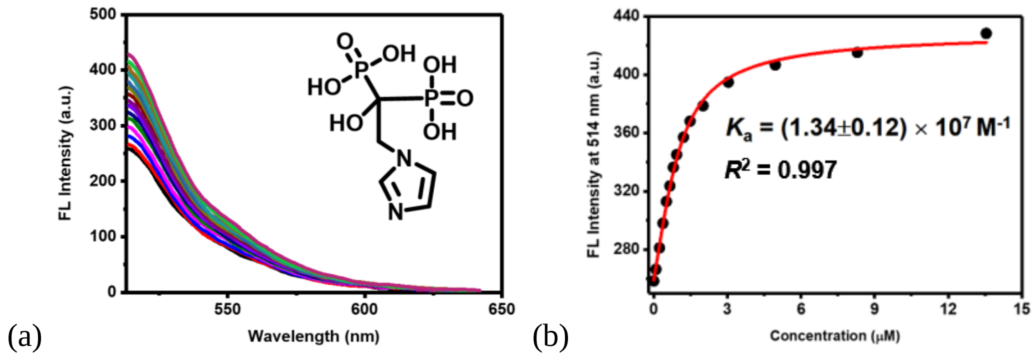

Figure S1: (a) Competitive fluorescence titration of GC5A·Fl (0.9/1.0 μM) with zoledronate (up to 13.6 μM), λ_{ex} = 500 nm. (b) The associated titration curve at λ_{em} = 514 nm and fit according to a 1:1 competitive binding model.

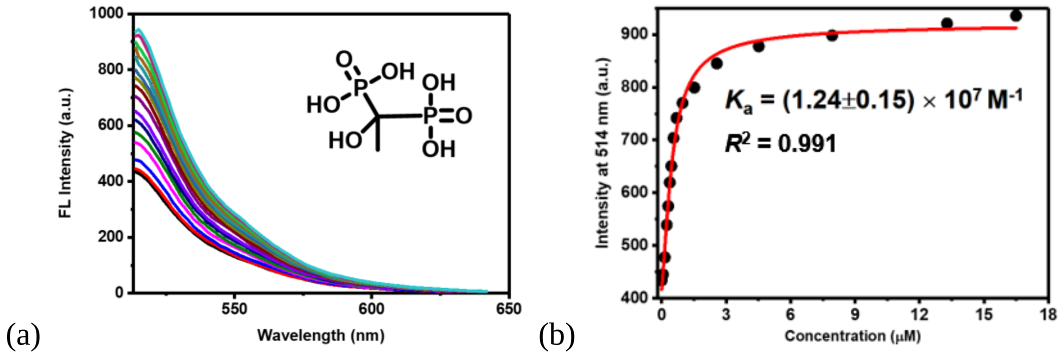

Figure S2: (a) Competitive fluorescence titration of GC5A·Fl (0.4/0.3 μM) with etidronate (up to 16.5 μM), λ_{ex} = 500 nm. (b) The associated titration curve at λ_{em} = 514 nm and fit according to a 1:1 competitive binding model.

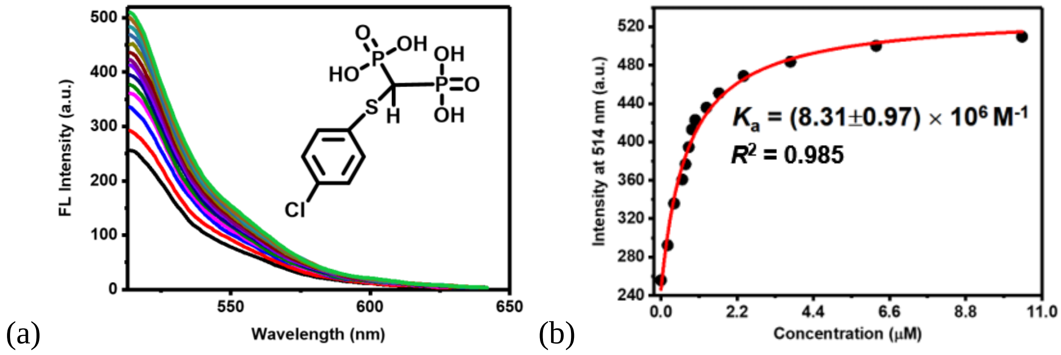

Figure S3: (a) Competitive fluorescence titration of GC5A·Fl (0.9/1.0 μM) with tiludronate (up to 10.4 μM), λ_{ex} = 500 nm. (b) The associated titration curve at λ_{em} = 514 nm and fit according to a 1:1 competitive binding model.

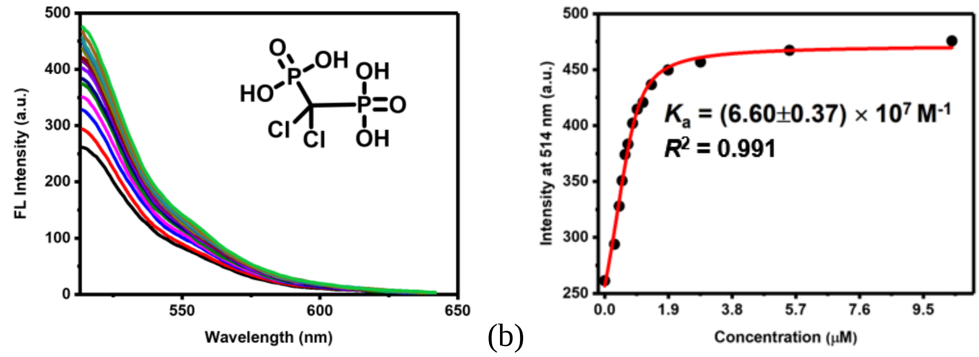

(a) (b)

Figure S4: (a) Competitive fluorescence titration of GC5A·Fl (0.9/1.0 μM) with clodronate (up to 10.4 μM), λ_{ex} = 500 nm. (b) The associated titration curve at λ_{em} = 514 nm and fit according to a 1:1 competitive binding model.

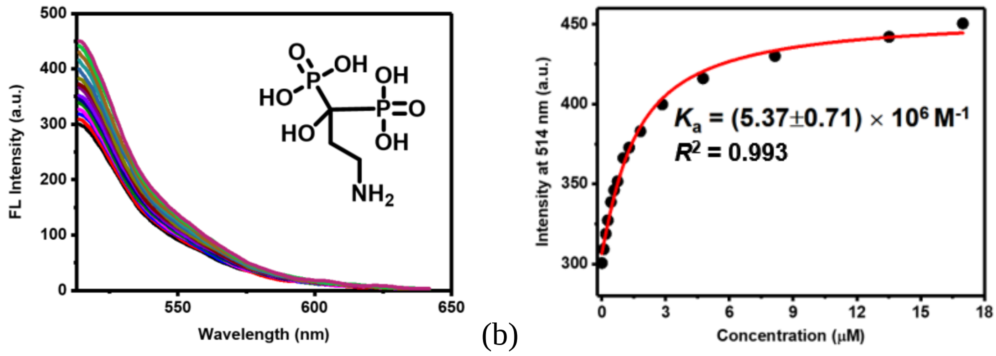

(a) (b)

Figure S5: (a) Competitive fluorescence titration of GC5A·Fl (0.9/1.0 μM) with pamidronate (up to 17.0 μM), λ_{ex} = 500 nm. (b) The associated titration curve at λ_{em} = 514 nm and fit according to a 1:1 competitive binding model.

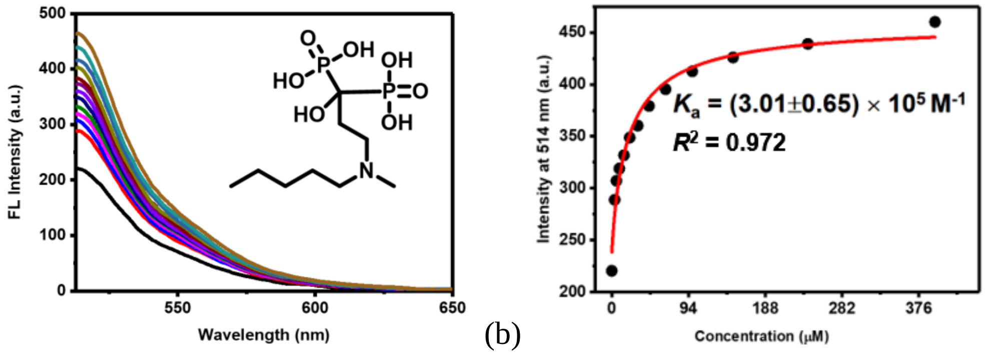

(a) (b)

Figure S6: (a) Competitive fluorescence titration of GC5A·Fl (0.9/1.0 μM) with ibandronate (up to 0.38 mM), λ_{ex} = 500 nm. (b) The associated titration curve at λ_{em} = 514 nm and fit according to a 1:1 competitive binding model.

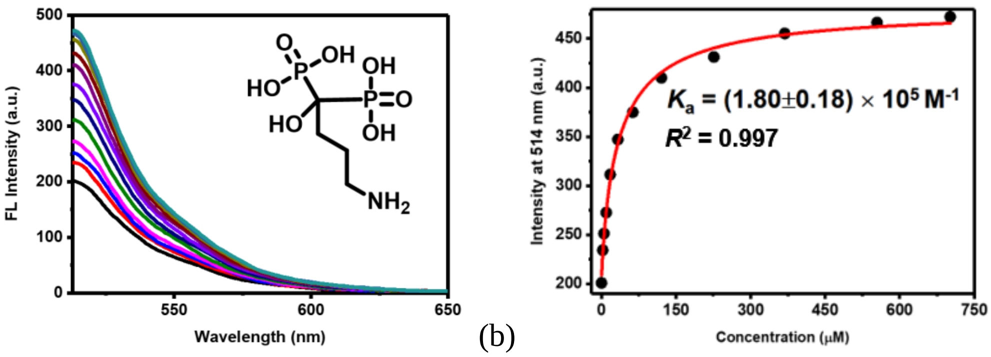

Figure S7: (a) Competitive fluorescence titration of GC5A·Fl (0.9/1.0 μM) with alendronate (up to 0.7 mM), λ_{ex} = 500 nm. (b) The associated titration curve at λ_{em} = 514 nm and fit according to a 1:1 competitive binding model.

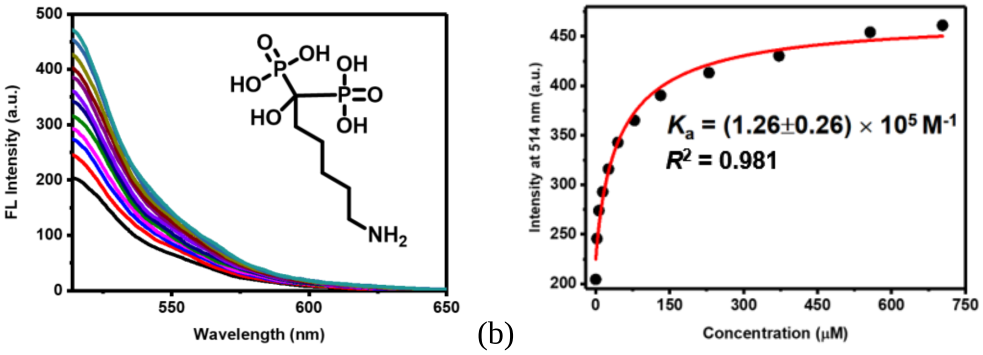

Figure S8: (a) Competitive fluorescence titration of GC5A·Fl (0.9/1.0 μM) with neridronate (up to 0.70 mM), λ_{ex} = 500 nm. (b) The associated titration curve at λ_{em} = 514 nm and fit according to a 1:1 competitive binding model.

References

1. Zheng, Z.; Geng, W.-C.; Gao, J.; Wang, Y.-Y.; Sun, H.; Guo, D.-S.. *Chem. Sci.* **2018,** *9*, 2087-2091.

2. Shmaefsky, B.R. *Am. Biol. Teach.* **1990,** *52*, 170-172.
